# Changes in Nursing Practice Among Clinical Nurses After Experiencing a Patient Safety Incident: Partial Least Squares Structural Equation Modeling

**DOI:** 10.1155/jonm/1587897

**Published:** 2025-03-04

**Authors:** Sunmi Kim, Seohee Jeong, Seok Hee Jeong

**Affiliations:** ^1^Department of Nursing, College of Nursing, Woosuk University, Wanju-gun, Republic of Korea; ^2^Medical Care Department, QI Team, Jeonbuk National University Hospital, Jeonju-si, Republic of Korea; ^3^Department of Nursing, College of Nursing, Research Institute of Nursing Science, Jeonbuk National University, Jeonju-si, Republic of Korea

**Keywords:** just culture, nurses, patient safety, second victim

## Abstract

**Background:** Nurses experiencing second victimization after a patient safety incident face challenges in developing effective coping strategies. Active coping can lead to constructive practice changes within a just culture. However, no theoretical model has yet tested the relationships among a just culture, second victim variables, coping strategies, and practice changes.

**Methods:** A nationwide online survey was conducted using proportional quota sampling based on region, representing 0.7% of nurses in tertiary hospitals across various Korean regions as of August 2022. Partial least squares structural equation modeling (PLS-SEM) was used to develop the hypothesized model, determine the model fit, and test research hypotheses. Descriptive statistics, model fit, and path analysis were performed using SPSS and Smart-PLS.

**Results:** The final analysis included 461 clinical nurses. Six significant pathways were identified: A just culture positively influenced constructive changes in nursing practice through second victim experience and avoidant coping (*B* = 0.07, *p* < 0.001). In the absence of a just culture, constructive changes decreased (*B* = −0.12, *p* < 0.001). The just culture negatively influenced defensive changes in nursing practice through second victim experience (*B* = −0.24, *p* < 0.001). The just culture negatively influenced defensive changes in nursing practice through second victim experience and avoidant coping (*B* = −0.10, *p* < 0.001). Without the just culture, defensive changes in nursing practice increased (*B* = 0.19, *p* < 0.001). The just culture reduced avoidant coping through second victim experience (*B* = −0.25, *p* < 0.001).

**Conclusions:** This study provides pathways to increase constructive nursing practice changes and decrease defensive nursing practice changes in nurses who have experienced a patient safety incident.

**Implications for Nursing Management:** The just culture needs to be established in a nursing practice setting and healthcare organizations. This study, using a representative sample through proportional quota sampling, provides reliable and valid evidence for nursing practice and healthcare organizations regarding the just culture, second victim experiences, and patient safety.

## 1. Introduction

Patient safety is a critical global health priority and a fundamental requirement for providing quality healthcare [1]. To ensure effective and safe healthcare, countries and healthcare institutions have implemented various measures to specialize in patient safety, including enacting patient safety laws and establishing patient safety management systems [2, 3]. Despite the implementation of such measures, unexpected patient safety incidents continue to occur. An average of 1689 patient safety incidents are reported every month in South Korea, and this number continues to rise [4]. Patient safety incidents that occur during the provision of healthcare services can result in major and minor adverse effects on patients, including death and disability [1, 4].

Patient safety incidents have an impact not only on patients but also on healthcare workers and organizations involved [5, 6]. It is crucial to consider the impact on all parties affected by patient safety incidents, including healthcare workers (referred to as second victims) and healthcare organizations (referred to as third victims), not just the patients who are the first victims. Healthcare workers who experience second victimization are crucial, as they provide medical and nursing care after patient safety incidents. Negative psychological experiences can have an impact not only on healthcare professionals themselves but also on their patients and organizations [7–10]. Second victim experiences occur when healthcare providers involved in patient safety incidents experience negative emotions or difficulties, both physically and mentally, in addition to the patients [7–9, 11]. Among healthcare providers, nurses have the most patient contact. Thus, patient safety incidents are prevalent in nurses, with 60.4% of clinical nurses experiencing them [9]. Patient safety incidents can cause psychological distress, fear, long-lasting negative emotions, and a loss of confidence among nurses [7, 8]. These negative effects can lead to intentions to leave the nursing field [9, 10]. Therefore, healthcare organizations must work to reduce the level of second victim experienced by nurses when unavoidable patient safety incidents occur, in addition to ongoing efforts to reduce the incidence of patient safety incidents. Previous research studies on the second victim phenomenon experienced by nurses have primarily focused on the correlation between safety-related organizational culture in healthcare institutions and second victim experiences. For example, previous studies have investigated the impact of patient safety culture on second victim distress, support needs, and outcomes such as turnover and absenteeism [10, 12]. Previous research has also investigated effects of nonpunitive responses to errors on second victim distress and support needs [13]. It is crucial to noting that nurses who experience patient safety incidents, even those resulting in second victims, should view them as opportunities for learning and improvement. Effectively managing and overcoming patient safety incidents require reduced second victim experiences, which can bring about constructive changes to personal and organizational lives. Therefore, it is important to view patient safety incidents as opportunities to make constructive changes that can improve nursing practice.

Jeong and Jeong [14] have conducted research on coping strategies used by nurses who have experienced second victims and found that these strategies can significantly affect their nursing practice. Types of changes in nursing practice that occur are determined by the nature of these coping behaviors. Nurses who engage in avoidant coping behaviors may exhibit defensive changes in nursing practice, such as losing confidence in patient care or considering leaving the profession [14, 15]. Conversely, proactive and positive problem-solving behaviors can lead to constructive changes in nursing practice. For instance, nurses can increase their vigilance and conduct more thorough reviews of records and procedures during patient care [14, 15]. This trend has been observed worldwide, as demonstrated by studies such as those conducted by Chard [16] and Karga et al. [17].

However, the extent of second victim experiences after patient safety incidents may be affected by the culture of the organization or department [10, 12, 13]. It is crucial to providing organizational and peer support for second victims to promote a just culture [18]. A just culture is a culture of trust that encourages members of an organization to share critical information related to healthcare safety [19]. A just culture takes a systems-based approach to building defenses that can prevent errors or mitigate their effects [19]. The focus is not on blaming individuals who make mistakes but rather on fostering a culture of accountability [20]. Therefore, a just culture is considered an essential component of a safety culture [21]. In response, many healthcare organizations are shifting from an approach of holding individuals accountable after a patient safety incident to a just culture approach. This shift involves analyzing root causes of incidents and implementing improvements rather than focusing on individual blame [2].

Jeong et al. [22] have found that a just culture can significantly impact distress related to second victims and nurses' support needs. Their study revealed that a just culture not only could directly influence second victim outcomes but also could indirectly affect these outcomes through levels of distress and support needs. This finding emphasizes the crucial role of a just culture in shaping nurses' experiences of second victim experiences in healthcare settings. However, empirical studies testing causal relationships between a just culture, second victim experiences, coping strategies, and changes in nursing practice within a theoretical framework are lacking. Previous studies such as those by Jeong et al. [22], Jeong and Jeong [14], Chard [16], and Karga et al. [17] only provided partial insights into each variable, which limited the generalizability of their findings. Therefore, this study aimed to address this gap in the existing literature by proposing an integrated model. The model aims to comprehend how nurses who have experienced second victims by patient safety incidents can encourage constructive changes and reduce defensive changes in their nursing practice. The objective of this study was to create efficient strategies with positive impact on both individual nurses and the healthcare organization as a whole.

### 1.1. Purpose

This study aimed to build a hypothetical model to determine the impact of a just culture perceived by nurses who experienced patient safety incidents on second victim experiences, coping strategies, and changes in nursing practice and to identify direct and indirect effects of these variables on changes in nursing practice.

### 1.2. Hypothetical Model

**Figure 1:**
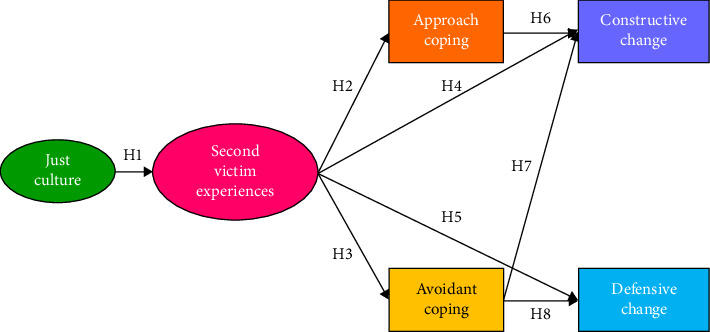
Conceptual framework.

For this study, a total of eight hypotheses were formulated as follows. The hypothesized model is shown in Figure 1.

  Hypothesis 1: A just culture has a significant impact on second victim experiences.

Existing studies have established patient safety culture as a key determinant in reducing second victim experiences among nurses [10, 12, 13]. In this context, the just culture plays a critical role. A just culture influences both the reporting of patient safety incidents and the trajectory of subsequent improvement, thereby influencing the presence and severity of second victim experiences [18]. A recent study of Korean nurses has also indicated both direct and indirect effects of a just culture on second victim experiences [22]. Consequently, this study posits that the influence of the just culture on second victim experiences manifests as a direct effect.

  Hypothesis 2: Second victim experiences have a significant impact on approach coping.
  Hypothesis 3: Second victim experiences have a significant impact on avoidant coping.

Individuals who experience second victims from patient safety incidents may engage in a variety of coping behaviors. These coping strategies patterns can vary among individuals. A meta-analysis of healthcare workers in different countries around the world has found that they mainly use task-oriented, emotion-oriented, and avoidance-oriented coping strategies [11]. In addition, a previous study of nurses at a tertiary general hospital in Korea has reported that effects of second victim experiences on approach coping and avoidant coping are statistically significant [14]. Therefore, this study posits that the effects of second victim experiences on approach coping and avoidant coping exert a direct influence.

  Hypothesis 4: Second victim experiences have a significant impact on constructive changes in nursing practice.
  Hypothesis 5: Second victim experiences have a significant impact on defensive changes in nursing practice.

A previous study of clinical nurses has found that second victim experiences have statistically significant effects on both constructive changes in nursing practice and defensive changes in nursing practice [14]. In addition, a meta-analysis has found that higher levels of trauma are associated with increased symptoms of depression, anxiety, and posttraumatic stress disorder among Koreans using avoidant coping [23]. Research has also shown that problem-focused coping has a positive effect on posttraumatic growth in individuals who have experienced trauma [24]. These findings suggest that coping styles used by nurses who have experienced second victims might lead to different changes in nursing practice. Therefore, the present study aimed to describe pathways through which second victim experiences could affect changes in nursing practice according to different coping styles and hypothesized that effects of second victim experiences on constructive changes in nursing practice and defensive changes in nursing practices were direct.

  Hypothesis 6: Approach coping has a significant impact on constructive changes in nursing practice.

Previous research involving nurses has identified certain coping strategies such as accepting responsibility, seeking social support, and engaging in planned problem-solving behaviors that can lead to constructive changes in nursing practice [14, 16, 17]. Therefore, this study posits that approach coping exerts a direct influence on constructive changes in nursing practice.

  Hypothesis 7: Avoidant coping has a significant impact on constructive changes in nursing practice.
  Hypothesis 8: Avoidant coping has a significant impact on defensive changes in nursing practice.

In a previous study of Korean nurses, avoidant coping had a significant negative effect on constructive changes in nursing practice and a significant positive effect on defensive changes in nursing practice [14]. Studies of nurses in other countries have also shown that avoidant coping behaviors can lead to changes in defensive practices [16, 17]. It has also been reported that people who have experienced trauma have higher levels of depression, anxiety, and posttraumatic stress disease when using avoidant coping [23]. Therefore, this study hypothesized that effects of avoidant coping on constructive changes and defensive changes in nursing practice were direct.

## 2. Methods

### 2.1. Study Design

This study used partial least square structural equation modeling (PLS-SEM) to develop a hypothesized model of nursing practice changes among Korean nurses who experienced patient safety incidents and to test the model's fit and research hypotheses.

#### 2.1.1. Sampling and Sample Size

This study targeted nurses currently employed in Korea. The proximate population was nurses working in a tertiary hospital in Korea. Selection criteria were: currently employed as a nurse in a tertiary hospital in Korea; working as a staff nurse in a ward or intensive care unit to provide direct patient care; having direct experience of at least one patient safety incident (near miss, adverse event, or sentinel event) within the past 6 months; and having at least 1 year of clinical experience. Nurses with a minimum of 1 year of clinical experience were chosen for this study because obtaining an accurate depiction of patient safety incidents with less than 1 year of clinical experience could be challenging.

Proportional quota sampling was employed to ensure a representative distribution of participants across the South Korean healthcare system. The quotas were based on the administrative districts of hospitals, with South Korea comprising a total of 17 administrative districts. The quota for each district was set at 0.7% of registered nurses, based on statistics from August 2022 [25]. The total sample size was 461, with the distribution across the 17 districts detailed in Supporting Table 1. The sample size of 461 participants falls within the optimal range for structural equation modeling, which generally recommends 200–400 participants for structural model analysis [26]. This sample size meets the recommended criterion, providing sufficient statistical power while maintaining practical feasibility during data collection.

#### 2.1.2. Data Collection

Data were collected from February to May 2023. The experience of patient safety incidents is a sensitive issue for individuals and organizations. Thus, it is necessary to ensure anonymity of respondents when conducting surveys. Therefore, this study was conducted online to ensure anonymity and reliability of the study. To recruit nurses working in tertiary general hospitals across 14 administrative districts, we requested participation in the study via email to regional nursing societies and hospital educators. We also promoted this study through social media. In addition, for reducing under-coverage, we utilized a multiplicity sampling strategy, such as snowball sampling, in which respondents were introduced to other research subjects or recommended. To decrease unit nonresponse rates, participants were given mobile coffee coupons as an incentive.

To ensure a representative sample, quota sampling was applied based on the number of participants allocated to each of the 17 administrative districts. Data collection was conducted through an online survey and continued until the target number of participants for each region was met. Once the target quota was reached, participation from that region was restricted. However, because of the nature of online surveys, simultaneous responses occurred, leading to an excess of participants in certain regions. Only the earliest responses up to the predetermined target quota were included in the final analysis. This resulted in a final sample size of 461 participants across all 17 administrative districts.

### 2.2. Measures

#### 2.2.1. Demographics

Sociodemographic characteristics included gender, marital status, religion, level of education, and region. Occupational characteristics included clinical career, type of work, and type of employment. Patient safety incident experiences included near miss, adverse event, and sentinel events.

#### 2.2.2. Just Culture

A just culture refers to a culture in which trust is established by encouraging and rewarding members of the organization to share critical information related to safety. There are clear distinctions between acceptable and unacceptable behaviors [19]. This study utilized the Just Culture Assessment Tool (JCAT) developed by Petschonek et al. [27] after it was translated into Korean. The tool underwent a translation process by a nursing professor fluent in both Korean and English, followed by a professional translator working for Editage who is also fluent in both languages. Content validity was then reviewed by three nursing professors. Finally, three nurses reviewed the instrument to ensure that the terminology was appropriate for their profession. The instrument utilized in this study was evaluated with a 7-point Likert scale with 6 subscales. This study utilized a reversed scoring system, with a higher score indicating a higher level of the just culture. Cronbach's alpha coefficient for all 27 items was 0.88.

#### 2.2.3. Second Victim Experience

Second victim experience refers to the phenomenon of healthcare providers experiencing negative emotions and symptoms because of patient safety incidents such as medical errors and adverse events [28]. In this study, second victim experience was measured using the Korean version of the Second Victim Experience and Support Tool (K-SVEST) [29], an adapted version of the Second Victim Experience and Support Tool (SVEST) [30]. The K-SVEST is a 5-point Likert scale consisting of three subscales: second victim distress, support needs, and second victim outcomes. The scale has a total of 28 items, with positive items being reversed. Higher scores indicated higher levels of second victim experience. In this study, Cronbach's alpha coefficients for the three subscales were 0.78, 0.71, and 0.77, respectively.

#### 2.2.4. Coping

This study measured coping through two methods: approach coping and avoidant coping. Approach coping involves taking appropriate actions in stressful situations or using changes in the situation to feel more in control. Avoidant coping involves avoiding anxiety-provoking stimuli, selective inattention, and ignoring warnings of danger [31]. This study utilized the Brief COPE instrument developed by Carver [32] in its Korean version [33], which was validated with Korean nurses [14]. The instrument comprised 28 items on a 4-point scale and included two subscales, approach coping and avoidant coping, with higher scores indicating greater use of coping behaviors in each domain. Cronbach's alpha coefficients of the two subscales were 0.80 and 0.82, respectively.

#### 2.2.5. Changes in Nursing Practice

This study defined changes in nursing practice as changes in nurses' attitudes toward nursing practice, specifically constructive or defensive changes, after experiencing patient safety incidents [16]. The “change in practice tool” was one of seven questionnaires included in the “Perioperative Nurse Questionnaire” developed by Chard [16]. The questionnaire was translated into Korean and validated with Korean nurses [14]. This tool is a 15-item, 4-point scale consisting of constructive changes in nursing practice and defensive changes in nursing practice, with higher scores indicating more changes in nursing practice in that area. Cronbach's alpha coefficients of the two subscales were 0.76 and 0.70, respectively.

### 2.3. Statistical Analysis

General characteristics of participants were analyzed by calculating means, standard deviations, frequencies, and percentages. The internal consistency reliability of instruments was assessed using Cronbach's alpha coefficient. Data were analyzed using SPSS version 23.0 (IBM Corp., Armonk, NY, USA). Categorical variables are expressed as percentages (%) and continuous variables are presented as mean ± standard deviation.

The SmartPLS program version 4.0.9.3 was used to conduct the analysis to test the hypothesized model. The just culture and second victim experiences were formative indicators among variables used in this study. Thus, a formative indicator model was used. To check the correlation between variables, Pearson's correlation coefficient was calculated. The measurement model and the structural model were evaluated for hypothesized models. The measurement model was evaluated using model fit and variance inflation factor (VIF). Model fit was assessed using standardized root mean square residual (SRMR), unweighted least squares discrepancy (^d^ULS), and geodesic discrepancy (^d^G). In the PLS model, a consistent PLS algorithm was considered appropriate if the SRMR was less than 0.08 and both ^d^ULS and ^d^G were less than 0.95 (or 0.99). If these conditions were not met, the PLS algorithm was used for the analysis [34]. The VIF value was used to check acceptance criteria of the external model. To evaluate the structural model of the hypothesis, we analyzed it using the path weighting scheme and resampled it 50,000 times using the bootstrapping method. The path weighting scheme is an estimation method that can maximize the *R*^2^ of latent endogenous variables. It can be applied to any model [35]. A structural model assesses the inner model by evaluating the VIF, coefficient of determination *R*^2^, effect size *f*^2^, and predictive relevance *Q*^2^. If the VIF value was less than 5, there was no multicollinearity among latent variables. The coefficient of determination *R*^2^ was evaluated as small for 0.25, medium for 0.50, and large for 0.75. The effect size *f*^2^ represents the relative influence of the exogenous variable on the endogenous variable. It indicates how much the exogenous variable contributes to the *R*^2^ of the endogenous variable [36, 37]. A small effect is indicated by an *f*^2^ value of 0.02. A medium effect is indicated by a value 0.15, and a large effect is indicated by a value of 0.35. The Stone-Geisser *Q*^2^ value was used to assess the predictive fit of a structural model. A *Q*^2^ value greater than zero indicated a good predictive fit of the exogenous latent variable to the endogenous variable [38–40].

### 2.4. Ethical Approval

Data for this study were collected through an online survey. The online survey screen was designed to confirm participant's consent prior to taking the survey. The survey was started only after participant's consent. This study was approved by the Institutional Review Board (IRB) of Jeonbuk National University (IRB approval number: 2023-01-019-002).

## 3. Results

### 3.1. General Characteristics of Study Participants

The average age of participants was 31.3 years. Of all participants, 89.2% were female. The majority (70.9%) were single. In addition, 61.6% had no religious affiliation. Regarding clinical experience, 31.9% had more than 3 to less than 5 years of experience. The majority (75.1%) had three shifts. In addition, 79.0% were employed full time, and 79.8% worked on a ward unit. A total of 96.1% of participants had experienced a near miss, 60.3% had experienced an adverse event, and 18.4% had experienced a sentinel event (Table 1).

### 3.2. Measurement Model

The goodness of fit of the saturated model analyzed by PLS and PLSc algorithm for the research model is shown in Supporting Table 2. As a result of the model fit test of PLSc, the SRMR was 0.10, which was more than 0.08. Thus, it was not suitable. The ^d^ULS was 0.99, which was not suitable because it was more than 0.95. The ^d^G was 0.18, which was less than 0.95. Since the PLSc model was not suitable, the research model used in this study was the PLS structural equation.

PLS analysis results for the outer model are shown in Supporting Table 3. VIF values were less than 5, suggesting acceptability. The just culture was significantly influenced by x1 (*p*=0.023), x3 (*p*=0.005), and x6 (*p*=0.006). Second victim experiences were significantly formed by x7 (*p*=0.003), x8 (*p* < 0.001), and x9 (*p* < 0.001).

Correlations between variables are shown in Table 2. The just culture was negatively correlated with second victim experiences (*r* = −0.40, *p* < 0.001), avoidant coping (*r* = −0.25, *p* < 0.001), and defensive changes in nursing practice (*r* = −0.26, *p* < 0.001), but positively correlated with approach coping (*r* = 0.22, *p* < 0.001) and constructive changes in nursing practice (*r* = 0.29, *p* < 0.001). Second victim experiences were positively correlated with avoidant coping (*r* = 0.50, *p* < 0.001) and defensive changes in nursing practice (*r* = 0.68, *p* < 0.001). Approach coping were positively correlated with avoidant coping (*r* = 0.25, *p* < 0.001), constructive changes (*r* = 0.53, *p* < 0.001), and defensive changes in nursing practice (*r* = 0.12, *p*=0.012). Avoidant coping was positively correlated with defensive changes in nursing practice (*r* = 0.61, *p* < 0.001).

### 3.3. Structural Models

Results of the PLS structural equation model are shown in Table 3. VIF values of the internal model ranged from 1.000 to 1.488, all of which were less than 5, indicating that there was no multicollinearity among latent variables. Model analysis showed that the coefficient of determination was 0.30 for second victim experiences, 0.03 for approach coping, 0.21 for avoidant coping, 0.33 for constructive changes in nursing practice with a weak contribution, and 0.53 for defensive changes in nursing practice with a moderate contribution. The value of *Q*^2^, which indicated the predictive fit, ranged from 0.02 to 0.27, all of which were greater than zero, indicating a good predictive fit. Therefore, the PLS structural equation model was considered adequate. Regarding the effect on second victim experiences, it was found that the just culture (*B* = −0.54, *p* < 0.001) had a significant effect on second victim experiences. The higher the just culture, the lower the second victim experiences, showing a significant effect with an explanatory power of 0.30 and an effect size of 0.42. Second victim experiences did not have a significant effect on approach coping (*B* = −0.19, *p*=0.075). For the effect on avoidant coping, second victim experiences had a significant effect on avoidant coping (*B* = 0.46, *p* < 0.001). Higher second victim experiences were associated with higher avoidant coping, with an explanatory power of 0.21 and an effect size of 0.27, indicating a moderate effect. In terms of effect on constructive changes in nursing practice, approach coping (*B* = 0.61, *p* < 0.001) and avoidant coping (*B* = −0.27, *p* < 0.001) were found to have significant effects on constructive changes in nursing practice. The higher the approach coping and the lower the avoidant coping, the higher the constructive changes in nursing practice, with an explanatory power of 0.33 and an effect size of 0.46 for approach coping and 0.07 for avoidant coping. Regarding the effect on defensive changes in nursing practice, second victim experiences (*B* = 0.44, *p* < 0.001) and avoidant coping (*B* = 0.41, *p* < 0.001) were found to have significant effects on defensive changes in nursing practice. Higher levels of second victim experiences and higher levels of avoidant coping were associated with higher levels of defensive change, with an explanatory power of 0.53 for defensive changes in nursing practice and an effect size of 0.32 for second victim experiences and 0.27 for avoidant coping, indicating a moderate effect.

**Figure 2:**
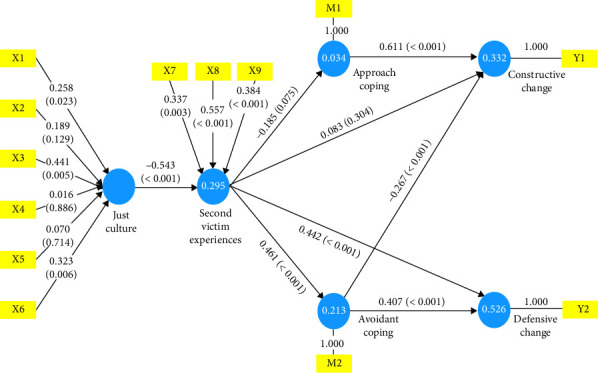
Results of partial least squares (PLS) structural equation model.

Results of an indirect effect test showed that the dual mediation effect of second victim experiences and avoidant coping on the effect of the just culture on constructive changes in nursing practice was significant (*B* = 0.07, *p* < 0.001). This indicates that a higher just culture will lead to lower second victim experiences, which can lead to lower avoidant coping, which then leads to higher constructive changes in nursing practice. In other words, a higher just culture can mediate second victim experiences and avoidant coping to increase the level of constructive changes in nursing practice. In addition, two out of five paths to constructive changes in nursing practice and three paths to defensive changes in nursing practice, which were dependent variables, were found to be significant. Specific results of the indirect effect test are shown in Table 3, Figure 2.

## 4. Discussion

To the best of our knowledge, this is the first study to present a theoretical model using PLS-SEM to explore the experiences of second victims following patient safety incidents and their impact on changes in nursing practice, based on a nationwide quota sampling approach. It differs from previous studies that have only examined a fragmentary relationship between related variables. By integrating organizational variables such as the just culture and nurses' personal variables such as coping strategies, we demonstrate comprehensive causal relationships that lead to changes in nursing practice. In addition, this study utilized a nationwide stratified sample of nurses from a tertiary general hospital in Korea, ensuring the sample's representativeness and the validity of study findings. The purpose of this study is to propose strategies for mitigating nurses' second victim experiences when unavoidable patient safety incidents occur and to provide positive feedback to healthcare organizations and individuals.

In this study, we found that two out of five paths to constructive changes in nursing practice and three paths to defensive changes in nursing practice were significant. Main findings are discussed below.

This study identified two significant pathways to constructive changes in nursing practice. The first pathway showed that second victim experiences increased avoidant coping, which in turn decreased constructive changes in nursing practice. This indicated that nurses who experienced patient safety incidents and had higher levels of second victim experiences engaged in more avoidant coping, resulting in decreased constructive changes in nursing practice. However, when only the just culture was added to the same pathway, it reduced second victim experiences, which in turn reduced avoidant coping but increased constructive changes in nursing practice. This finding suggests that a positive perception of the just culture within an organization can increase constructive changes in nursing practice by reducing second victim experiences and avoidant coping. It is important to note that the presence of the just culture is statistically significantly related to an increase or decrease of constructive changes in nursing practice, indicating that the just culture is a necessary precursor to constructive changes in nursing practice. The significance of this study lies in its empirical verification that the formation of a just culture in a healthcare organization can lead to constructive changes in nursing practice by reducing suffering from second victims and avoidant coping. Previous studies have shown that positive responses from senior staff after an event [17] and finding supportive resources and planning for problem-solving [16] are significantly associated with constructive changes in nursing practice. Furthermore, a systematic review of the just culture has revealed that trust is more effectively fostered through close relationships between managers and nurses, as opposed to organizational cultures that do not prioritize such relationships [41]. Taken together, results of this study and previous studies suggest that nurses working in healthcare organizations with established just culture may experience reduced levels of avoidant coping even when they experience patient safety incidents because of organizational efforts to communicate with each other and work together to improve problems based on trust, leading to constructive changes in nursing practice.

This study examined three significant pathways to defensive changes in nursing practice. Firstly, it was found that second victim experiences increased avoidant coping, which in turn increased defensive changes in nursing practice. This suggests that nurses who experience second victims because of patient safety incidents are more likely to engage in defensive changes in nursing practice by engaging in avoidant coping. Interestingly, the addition of the just culture to the pathway resulted in decreased defensive changes in nursing practice. Specifically, the just culture decreased second victim experiences, which in turn decreased avoidant coping, leading to decreased defensive changes in nursing practice. Furthermore, the pathway in which the just culture bypassed avoidant coping through second victim experiences and directly reached defensive changes in nursing practice was found to be significant. Higher levels of the just culture were associated with decreased second victim experiences, which in turn led to decreased defensive changes in nursing practice. This study confirmed that nurses working in organizations without the just culture might experience patient safety incidents that could lead to defensive changes in nursing practice. However, nurses working in organizations with established just culture experienced less defensive changes in nursing practice, even if they experienced second victims from patient safety incidents. Therefore, it is important to establish a just culture as an organizational culture in healthcare organizations before, rather than after patient safety incidents. A previous study of nurses who have experienced medication errors [42] has found that nurses report feeling uncomfortable disclosing errors, which can increase the burden of the reporting process. In addition, negative reactions from senior staff [17] and assignment of responsibility for errors [16] have also been reported to lead to defensive changes in nursing practice. A systematic review of quantitative, qualitative, and mixed studies on nurses' barriers to reporting medication errors and near misses has found that nurses are hesitant to report errors because of their fear of being blamed or perceived as troublemakers by colleagues or managers [43]. Therefore, it is necessary for healthcare organizations to make organizational efforts to reduce the level of second victim experiences among nurses who suffer from stress and anxiety [18]. A systematic review of the just culture has confirmed that when manager monitors employees, they tend to cover up or hide mistakes [41]. Healthcare organizations should establish multidisciplinary collaboration to establish a just culture. This includes sharing preventive measures and improvement plans with all members of the organization and facilitating smooth and supportive communication in an open and formal setting in response to patient safety incidents.

This study found that the establishment of a just culture in an organization preceded increased constructive changes in nursing practice and decreased defensive changes in nursing practice among nurses experiencing patient safety incidents. In this study, it was found that the just culture reduced negative effects of second victim experiences by decreasing nurses' exposure to them. The ways in which the just culture accomplishes this are discussed in more detail below.

This study found that the just culture had a direct and significant effect on second victim experiences. Specifically, nurses who perceived their organization's just culture more positively reported lower second victim experiences. This finding is consistent with a previous study conducted in South Korea examining the relationship between quality of work-related life and the just culture among 622 nurses [44]. That study found that nurses who perceived higher levels of organizational just culture reported higher quality of work-related life, particularly among those who experienced patient safety incidents [44]. It supports the idea that a nonpunitive approach to errors is beneficial for nurses who experience second victims. In addition, nurses are less likely to report errors if they are handled negatively, as found in a study by Caliban and Kynoch [7]. Quillivan et al. [12] has also found that a nonpunitive response to errors, measured by the patient safety culture tool, is a significant predictor of reduced stress because of second victims, which aligns with findings of the present study. High levels of the just culture in an organization can reduce second victim experiences, even in the face of patient safety incidents. To establish the just culture and reduce nurses' second victim experiences, overall support from the healthcare organization is required, including trust between managers and employees, as well as support from peers and the nursing administration [18].

However, an integrative review by White and Delacroix [18] has found that programs and support services for employees who experience second victims after patient safety incidents are lacking. This suggests that a successful just culture requires efforts to build trust among staff and establish a support system for healthcare workers who experience second victims. To clarify the causal relationship between the just culture and second victim experiences, a study of nurses in a tertiary general hospital has suggested direct and indirect pathways through which the just culture has a significant impact in reducing second victim experiences [22]. The implementation of the just culture in an organization is influenced by factors such as communication about events, continuous improvement, and trust [27]. Therefore, it is believed that in organizations with a well-established just culture, which includes open reporting of errors and multifaceted efforts for patient safety, constructive changes in nursing practice will increase and defensive changes in nursing practice will decrease. This is because of increased organizational support and efforts for improvement. Several countries such as South Korea have recently conducted research and made systematic efforts to institutionalize just culture [22, 45]. To establish and institutionalize a just culture in healthcare organizations, having a clear understanding of the concept is crucial. Executives and managers should focus on improving the system without blaming individuals, while members of the healthcare organization should strive to fulfill their individual responsibilities. This will build mutual trust and ensure that a balanced just culture is established in the right direction.

### 4.1. Limitations

This study was limited to nurses working in tertiary general hospitals in South Korea, which might affect its generalizability because of potential sociocultural differences in medical practices and contexts in non-Korean settings. Therefore, caution is needed when extrapolating these findings to nursing populations in different cultural or institutional environments. Furthermore, this investigation did not differentiate patient safety incidents based on their severity. A correlation between the intensity of second victim experiences and the nature of the incident, whether it is a near miss, an adverse event, or a sentinel event, is hypothesized. Future research should aim to delineate the ability of the just culture to reduce second victim experiences depending on the specific typology of the encountered event and its subsequent influence on coping strategies and practice modifications.

## 5. Conclusions

This study demonstrates that the just culture reduces second victim experiences among nurses who encounter patient safety incidents. The reduction of second victim experiences decreases avoidant coping, thereby increasing constructive changes in nursing practice and reducing defensive changes. In addition, approach coping among nurses directly promotes constructive changes in nursing practice. These findings highlight the critical role of implementing a just culture within healthcare organizations to foster positive changes in nursing practice and minimize defensive behaviors. Establishing a just culture is crucial for achieving meaningful improvements in organizational trust and patient safety.

Based on the results of this study, the following suggestions are made. First, it is recommended to educate about the importance of the just culture and to make efforts to establish the just culture in nursing practice. Second, the model needs to be tested with subjects from various international contexts to confirm its universal applicability in the future. Finally, it is recommended to investigate factors that can influence approach coping in nurses who have experienced second victims. This will enrich and broaden the applicability of the model.

## Figures and Tables

**Table 1:** General characteristics of study participants (*N* = 461).

Characteristics	Categories	*n* (%)	M±SD (range)
*Sociodemographic characteristics*			
Gender	Male	50 (10.8)	
	Female	411 (89.2)	

Age (years)	< 30	221 (47.9)	31.3 ± 5.32
	30 ∼ < 40	197 (42.7)	(23 to 50)
	≥ 40	43 (9.4)	

Marital status	Single	327 (70.9)	
	Married	134 (29.1)	

Religion	No	284 (61.6)	
	Christianity	108 (23.4)	
	Catholicism	41 (8.9)	
	Buddhism	27 (5.9)	
	Others	1 (0.2)	

Level of education	College	27 (5.9)	
	University	366 (79.4)	
	Graduate school	32 (6.9)	
	≥ Master's degree	36 (7.8)	

Region	Seoul capital city	183 (39.7)	
	Busan city	28 (6.1)	
	Daegu city	43 (9.3)	
	Incheon city	30 (6.5)	
	Gwangju city	16 (3.5)	
	Daejeon city	10 (2.2)	
	Ulsan city	11 (2.4)	
	Gyeonggi-do	52 (11.3)	
	Gangwon-do	14 (3.0)	
	Chungcheongbuk-do	7 (1.5)	
	Chungcheongnam-do	19 (4.1)	
	Jeollabuk-do	18 (3.9)	
	Jeollanam-do	5 (1.1)	
	Gyeongsangnam-do	25 (5.4)	

*Occupational characteristics*			
Clinical career (years)	1 to < 3	112 (24.3)	6.05 ± 4.98
	3 to < 5	147 (31.9)	(1 to 30)
	5 to < 10	119 (25.8)	
	≥ 10	83 (18.0)	

Type of work	Nonshift	65 (14.1)	
	2-shift	50 (10.8)	
	3-shift	346 (75.1)	

Type of employment	Full time	364 (79.0)	
	Contract	97 (21.0)	

Work unit	Ward	368 (79.8)	
	ICU	93 (20.2)	

Patient safety incident experiences			
Near miss	No	18 (3.9)	
	Yes	443 (96.1)	
Adverse event	No	183 (39.7)	
	Yes	278 (60.3)	
Sentinel event	No	376 (81.6)	
	Yes	85 (18.4)	

Abbreviations: ICU, intensive care unit; M, mean; SD, standard deviation.

**Table 2:** Means, standard deviations, internal consistencies, and correlations of study variables.

Variables	M	SD	*α*	JC	SVEs	APC	AVC	CCNP	DCNP
				*r* (*p*)	*r* (*p*)	*r* (*p*)	*r* (*p*)	*r* (*p*)	*r* (*p*)
JC	4.40	0.73	0.88	1					
SVEs	3.00	0.43	0.83	−0.40 (< 0.001)	1				
APC	2.61	0.47	0.80	0.22 (< 0.001)	−0.07 (0.121)	1			
AVC	2.16	0.53	0.82	−0.25 (< 0.001)	0.50 (< 0.001)	0.25 (< 0.001)	1		
CCNP	3.03	0.44	0.76	0.29 (< 0.001)	−0.05 (0.247)	0.53 (< 0.001)	−0.08 (0.101)	1	
DCNP	2.56	0.55	0.70	−0.26 (< 0.001)	0.68 (< 0.001)	0.12 (0.012)	0.61 (< 0.001)	0.09 (0.046)	1

Abbreviations: APC, approach coping; AVC, avoidant coping; CCNP, constructive change in nursing practice; DCNP, defensive change in nursing practice; JC, just culture; M, mean; SD, standard deviation; SVE, second victim experiences.

**Table 3:** Direct and indirect effect analyses for the hypothetical model.

**Direct effect**	***B***	***M***	**SD**	***t***	***p***	**VIF**	**f** ^2^	**R** ^2^ **(adj** **R** ^2^ **)**	**Q** ^2^	**Hypothesis**

JC ⟶ SVEs	−0.54	−0.55	0.06	9.37	< 0.001	1.000	0.42	0.30 (0.29)	0.27	H1: supported
SVEs ⟶ APC	−0.19	−0.17	0.10	1.78	0.075	1.000	0.04	0.03 (0.03)	0.02	H2: not supported
SVEs ⟶ AVC	0.46	0.46	0.06	8.25	< 0.001	1.000	0.27	0.21 (0.21)	0.04	H3: supported
SVEs ⟶ CCNP	0.08	0.09	0.08	1.03	0.304	1.446	0.01	0.33 (0.33)	0.02	H4: not supported
APC ⟶ CCNP	0.61	0.61	0.04	14.57	< 0.001	1.213	0.46			H5: supported
AVC ⟶ CCNP	−0.27	−0.27	0.05	5.35	< 0.001	1.488	0.07			H6: supported
SVEs ⟶ DCNP	0.44	0.44	0.06	7.77	< 0.001	1.270	0.32	0.53 (0.52)	0.07	H7: supported
AVC ⟶ DCNP	0.41	0.40	0.05	8.74	< 0.001	1.270	0.27			H8: supported

**Indirect effect**					***B***		***M***	**SD**	***t***	***p***

JC ⟶ SVEs ⟶ AVC ⟶ CCNP					0.07		0.07	0.02	4.34	< 0.001
JC ⟶ SVEs ⟶ APC ⟶ CCNP					0.06		0.06	0.04	1.65	0.099
JC ⟶ SVE ⟶ CCNP					−0.05		−0.05	0.04	1.10	0.270
SVEs ⟶ AVC ⟶ CCNP					−0.12		−0.13	0.03	3.85	< 0.001
SVEs ⟶ APC ⟶ CCNP					−0.11		−0.10	0.06	1.80	0.071
JC ⟶ SVEs ⟶ AVC ⟶ DCNP					−0.10		−0.10	0.02	6.56	< 0.001
JC ⟶ SVEs ⟶ DCNP					−0.24		−0.24	0.03	7.44	< 0.001
SVEs ⟶ AVC ⟶ DCNP					0.19		0.19	0.02	7.99	< 0.001
JC ⟶ SVEs ⟶ AVC					−0.25		−0.25	0.03	8.19	< 0.001
JC ⟶ SVEs ⟶ APC					0.10		0.10	0.06	1.62	0.105

*Note: B*: unstandardized regression coefficients; *f*^2^: effect size; *R*^2^: coefficient of determination; *Q*^2^: predictive relevance.

Abbreviations: APC, approach coping; AVC, avoidant coping; CCNP, constructive change in nursing practice; DCNP, defensive change in nursing practice; JC, just culture; M, mean; SD, standard deviation; SVE, second victim experiences; VIF, variance inflation factor.

## Data Availability

The data that support the findings of this study are available from the corresponding author upon reasonable request.

## Supporting Information

Supporting Table 1: Proportional quota sampling; distribution across the 17 administrative districts. Supporting Table 2: Model fit. Supporting Table 3: Outer model.
