# Supplementary material for: Changes in Nursing Practice Among Clinical Nurses After Experiencing a Patient Safety Incident: Partial Least Squares Structural Equation Modeling
Source: J Nurs Manag. 2025 Mar 4;2025:1587897. doi: 10.1155/jonm/1587897 (PMC11985243; doi:10.1155/jonm/1587897)
Supplement: Supporting Information — Additional supporting information can be found online in the Supporting Information section. [file 1587897.f1.docx]

Supplementary Table 1. Proportional quota sampling; Distribution across the 17 Administrative Districts

| Region | | Population  (Registered Nurses in tertiary hospitals) | | Sample Size  (0.7% of Population) | | Number of Participants in Study | |
| --- | --- | --- | --- | --- | --- | --- | --- |
| Seoul Capital City | | 26,132 | | 182.9 | | 183 | |
| Busan City | | 4,002 | | 28.0 | | 28 | |
| Daegu City | | 6,138 | | 43.0 | | 43 | |
| Incheon City | | 4,283 | | 30.0 | | 30 | |
| Gwangju City | | 2,327 | | 16.3 | | 16 | |
| Daejeon City | | 1,387 | | 9.7 | | 10 | |
| Ulsan City | | 1,601 | | 11.2 | | 11 | |
| Sejong City | | 0 | | 0 | | 0 | |
| Gyeonggi-do | | 7,388 | | 51.7 | | 52 | |
| Gangwon-do | | 2,070 | | 14.5 | | 14 | |
| Chungcheongbuk-do | | 936 | | 6.6 | | 7 | |
| Chungcheongnam-do | | 2,733 | | 19.1 | | 19 | |
| Jeollabuk-do | | 2,553 | | 17.9 | | 18 | |
| Jeollanam-do | | 645 | | 4.5 | | 5 | |
| Gyeongsangbuk-do | | 0 | | 0 | | 0 | |
| Gyeongsangnam-do | | 3,581 | | 25.1 | | 25 | |
| Jeju-do | | 0 | | 0 | | 0 | |
| Total | | 65,776 | | 460.432 | | 461 | |

Supplementary Table 2. Model fit

|  | 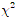 | SRMR | 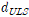 | 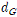 |
| --- | --- | --- | --- | --- |
| PLS | 535.096 | 0.13 | 1.43 | 0.23 |
| PLSc | 406.688 | 0.10 | 0.99 | 0.18 |
| Criteria |  | <0.08 | <0.95 | <0.95 |

Supplementary Table 3. Outer Model

| Measurement | Latent | weighted | M | SD | t | p | VIF |
| --- | --- | --- | --- | --- | --- | --- | --- |
| x1 | Just culture | 0.26 | 0.27 | 0.11 | 2.28 | 0.023 | 1.311 |
| x2 |  | 0.19 | 0.17 | 0.13 | 1.52 | 0.129 | 1.919 |
| x3 |  | 0.44 | 0.45 | 0.16 | 2.83 | 0.005 | 1.659 |
| x4 |  | 0.02 | 0.01 | 0.11 | 0.14 | 0.886 | 1.915 |
| x5 |  | 0.07 | 0.04 | 0.19 | 0.37 | 0.714 | 2.198 |
| x6 |  | 0.32 | 0.31 | 0.12 | 2.75 | 0.006 | 2.058 |
| x7 | Second victim experiences | 0.34 | 0.34 | 0.11 | 3.00 | 0.003 | 1.675 |
| x8 |  | 0.58 | 0.55 | 0.16 | 3.61 | <0.001 | 1.123 |
| x9 |  | 0.38 | 0.38 | 0.08 | 4.58 | <0.001 | 1.758 |
| M: Mean; SD: Standard deviation; VIF: Variance Inflation Factor. | | | | | | | |
